# Binge eating symptoms prevalence and relationship with psychosocial factors among female undergraduate students at Palestine Polytechnic University: a cross-sectional study

**DOI:** 10.1186/s40337-019-0263-1

**Published:** 2019-10-02

**Authors:** Manal M. Badrasawi, Souzan J. Zidan

**Affiliations:** Department of Nutrition and Food technology, Faculty of Agriculture and Veterinary Medicine, An-Najah National University, Tulkarm, West Bank, PO Box 7, Palestine

**Keywords:** Binge eating disorder, Prevalence, Risk factors, University students, Depression

## Abstract

**Background:**

Eating disorders pose a serious challenge to health services due to psychosocial and medical problems. Binge eating disorder (BED) is characterized as a pattern of overeating episodes followed by shame, distress and guilty feelings. Among eating disorders, BED has the highest prevalence, especially among females. The literature reported that BED is associated with nutritional status, socio-demographic factors, and psychological factors in different countries. This study aims to examine the prevalence of binge eating symptoms and its relationship with selected variables (i.e. socio-demographics, nutritional status and dietary habits).

**Methods:**

One hundred fifty-four female undergraduate students, from three different faculties at Palestine Polytechnic University, participated in the study. All the students who consented to join the study were assessed in terms of weight status using body mass index, dietary habits and medical profile. The screening for presence of binge eating symptoms was done using BEDS-7. The psychosocial factors were assessed by validated Arabic version of DASS-21.

**Results:**

Half of the participants (50%) had binge eating symptoms. No association between binge eating symptoms and socio-demographic variables was found. Similarly, binge eating symptoms was not related to body weight status, however, it was associated with eating between meals and number of snacks. A significantly higher score on depression, stress and anxiety was found among binge eaters than non-binge eaters.

**Conclusion:**

It was concluded that binge eating symptoms have considerable prevalence among the study participants, and it was significantly correlated with psychosocial factors. Future studies are needed to examine other risk factors and correlations. Educational programs are also recommended to increase the awareness of eating disorders as well as to promote healthy eating patterns.

## Plain English summary

Binge eating is an eating disorder characterized by frequent episodes of out-of-control eating large quantities of food (often very quickly and to the point of discomfort) followed by shame, distress and guilty feelings. The prevalence of binge is the highest as compared to other eating disorders. There is evidence that there is association between binge eating symptoms and nutritional status, social factors, self-esteem, depression, anxiety and stress. The outcomes of the current study showed that half of the female participants have experienced binge eating symptoms. There was also a significant relationship between psychosocial factors and binge eating symptoms.

## Introduction

Eating disorders are a group of mental disorders recognized by abnormal eating habits [1]. These disorders most often occur during the late stage of adolescence or early adulthood, and are associated to the social, physical, and psychological maturation of adolescents [2]. These disorders involve anorexia nervosa (AN), bulimia nervosa (BN), and binge eating disorder. AN is characterized by extreme weight loss, irrational fears of weight gain and obesity, and poor body image, whereas BN is known as a repeated bouts of uncontrolled, quick consumption of large amounts of food followed by self-induced vomiting, diuretics or laxative use, fasting, or vigorous exercise in order to avoid weight gain [3]. The focus of this research is on binge eating disorder (BED). The DSM-5 criteria since 2013 defined binge eating episode as a disorder occurs, on average, at least once per week for the past 3 months [4]. An episode of binge eating is recognized by eating abnormally large amounts of food over a limited period of time while experiencing feelings of loss of control [4]. Table 1 summarizes the diagnostic criteria for binge eating disorder.

**Table 1:** Diagnostic criteria for binge eating disorder ^a^

A. Recurrent episodes of binge eating, an episode being characterized by:	
1) Eating, in a discrete period of time (e.g., in any 2-h period), an amount of food that is definitely larger than most people would eat during a similar period of time	
2) A sense of lack of control during the episodes, e.g., a feeling that one can’t stop eating or control what or how much one is eating	
B. During most binge episodes, at least three of the following behavioural indicators of loss of control:	
1) Eating much more rapidly than usual	
2) Eating until feeling uncomfortably full	
3) Eating large amounts of food when not feeling physically hungry	
4) Eating large amounts of food throughout the day with no planned mealtimes	
5) Eating alone because of being embarrassed by how much one is eating	
6) Feeling disgusted with oneself, depressed, or feeling very guilty after overeating	
C. Marked distress regarding binge eating.	
D. The binge eating occurs, on average, at least once per week for the past 3 months.	
E. The binge eating is not associated with the recurrent use of inappropriate compensatory behavior as in bulimia nervosa and does not occur exclusively during the course of bulimia nervosa or anorexia nervosa	

^a^ Reprinted with permission from the *Diagnostic and Statistical Manual of Mental Disorders*, Fifth Edition (©2013). American Psychiatric Association [1]

Binge eating disorder seems to be the most common eating disorder, with estimates of the lifetime prevalence of binge eating disorder ranging from 1.9 to 2.8% [5] and according to some studies, it was found that binge eating disorder is more prevalent among women than males [6], this higher prevalence in males may be explained by males explicate binge eating symptoms in different way as compared to females [7]. Hudson and his colleagues found that there was no gender differences in the prevalence of subthreshold BED and binge eating behavior [8].. It also appears to be more prominent among overweight samples (30%) than among community samples (5% of females and 3% of males). In a college-student sample, the rate of binge eating disorder was 2.6% [3, 9]. This disorder is often linked with obesity even though a considerable rate of individuals (17–30%) have normal body weight [9].

The etiology of binge eating disorder is multifactorial. Cultural and social impacts are defined as one of the risk factors associated with binge eating [10]. Besides it is confirmed that individual’s with eating disorders can suffer from mental problems such as alcohol dependence, depression, social stress, daily activity-related stress and other anxiety issues [10]. Former studies have found that most individuals with binge eating experience higher rates of depression than normal individuals [10]. Other research has found that people with binge eating often suffer from several types of anxiety disorders [11].

Binge eating disorder is accompanied by multiple comorbidities including; psychiatric and medical comorbidities, and a higher mortality rates in comparison to subjects without eating disorders [12]. Moreover, binge-eaters are at a higher risk of developing dyslipidemia, hypertension, type 2 diabetes and metabolic syndrome compared with individuals who are not experiencing eating disorders. They may also have higher rates of sleeping problems when compared to subjects without eating disorders [12]. Psychiatric comorbidities are further related to binge eating disorder. Another study has found, 10 out of 14 studies confirmed a link between depression and binge eating disorder [13]. Former studies have noted that about 30–80% of binge-eaters have lifetime comorbid anxiety or mood disorders. Other personality problems and psychiatric comorbidities might be found in subjects with binge eating disorder including substance abuse, bipolar disorder, and gambling problems, as well as borderline personality disorders, avoidant, and obsessive-compulsive [12].

According to a recent study performed at Palestine, the prevalence of disordered eating attitudes is considerably high among female Palestinian university students [14]. In other research, it was reported that the rate of females at risk of eating disorders in Palestine was estimated to be 38.9% [15]. This could be an indication of the prevalence of binge eating symptoms among female university students in Palestine. However, no reliable research exist about the prevalence of binge eating symptoms among university female students.

To our knowledge, no study has yet assessed the prevalence of binge eating symptoms among female university students. The findings of the present study will add to the literature on binge eating symptoms among Arab female adolescents and young adults, which will help inform the design of educational programs to increase the undergraduate students’ awareness on eating disorders to promote healthy eating styles among them and the entire community as well. Furthermore, this study will determine the association between the presence of binge eating symptoms with depression, anxiety and stress among undergraduate students.

## Methods

### Study design

This study utilized a cross sectional design and aimed to determine the prevalence of binge eating symptoms among female undergraduate students in Palestine Polytechnic university- Hebron – West bank, Palestine, and to determine the relationship between binge eating symptoms and psychosocial factors. The study participants were selected from the three faculties in Palestine polytechnic University (Engineering, Applied Science and human Sciences). Participants were recruited by convenience sampling after personal invitation from the research team. The sample size was determined using Cochran formula for sample size calculation in a survey research [16]. The inclusion criteria included female participants who are doing their undergraduate degree in Palestine Polytechnic University. Participants were excluded if their age is less than 18 years old, have chronic diseases which can affect their dietary intake or nutritional status and participants who were pregnant during the data collection.

### Data collection and research tools

The data collection started in March 2018–May 2018. All participants were briefed about the study design and objectives, and they were informed about the type of data that would be collected, with affirmation on the optional participation. Participants who agreed to sign the consent form were included in the data collection. The local ethics committee of Palestine Polytechnic University approved and supported the current study.

The collected data included socio demographic characteristics; age, area of living, university discipline, academic achievement and self-reported medical history and smoking. Screening for binge eating symptoms was done using Binge Eating Disorder Screener-7 (BEDS-7) for use with adults. The BEDS-7 is a self-report screening tool that is designed to screen for BED symptoms rather than to make a diagnosis. It has been validated against DSM-5 diagnostic criteria [17]. BEDS-7 consists of 7 items asking about episodes of overeating during the last three months and the feelings after these episodes. Depending on the answers, participants are categorized into two categories (presence of binge eating symptoms or normal) following the suggested algorithms. The psychometric properties of BEDS-7; 100% sensitivity and 38.7% specificity [17]. The participants body mass index was assessed using anthropometric measurement (weight and height) following the standard methods reported by Lee and Nieman [18]. The measurements were measured in duplicate then the mean was recorded. The body mass index was calculated from the weight and height then categorized according to WHO cut off points [19]. Dietary intake was assessed using a validated food frequency questionnaire [20]. The Arabic version of the questionnaire consists of a total of ninety-eight food types in thirteen food groups. These foods correspond to items consumed in the Mediterranean region in general and in Palestine in particular. Nutrisuvey software was used to analyze the nutrients content of the selected foods to determine the intake. Participants were asked to answer the frequency of the consumption in addition to describe the portion size of the reported food [20]. The psychological parameters were assessed using the validated Arabic version of Depression Anxiety Stress Scales (DASS). The short form of DASS composed of 21 instrument measuring current (“over the past week”) symptoms of depression, anxiety, and stress. Participants were asked to use a 4-point combined severity/frequency scale to rate the extent to which they have experienced each item over the past week. The scale ranges from 0 (did not apply to me at all) to 3 (applied to me very much, or most of the time). Scores for depression, anxiety, and stress were calculated by summing the scores for the relevant items [21].

### Statistical analysis

All statistical analysis was carried out using the Statistical Package for Social Sciences (SPSS) software version 22. An alpha level of (0.05) was considered for all the statistical tests used in the study. Two-sided *p* values of (0.05) and (80%) power were statistically significant. The data were analyzed according to variable types. The descriptive analysis for the prevalence of presence of binge eating symptoms was done by calculating the frequencies and percentages. The association between the incidences was analyzed using Chi square tests because the variables are of categorical type including area of living, faculty, marital status and body mass index. The mean difference between the groups was done either by independent t-test (depression, anxiety, stress and diet intake).

## Results

### Subject Charactersitics

**Fig. 1:**
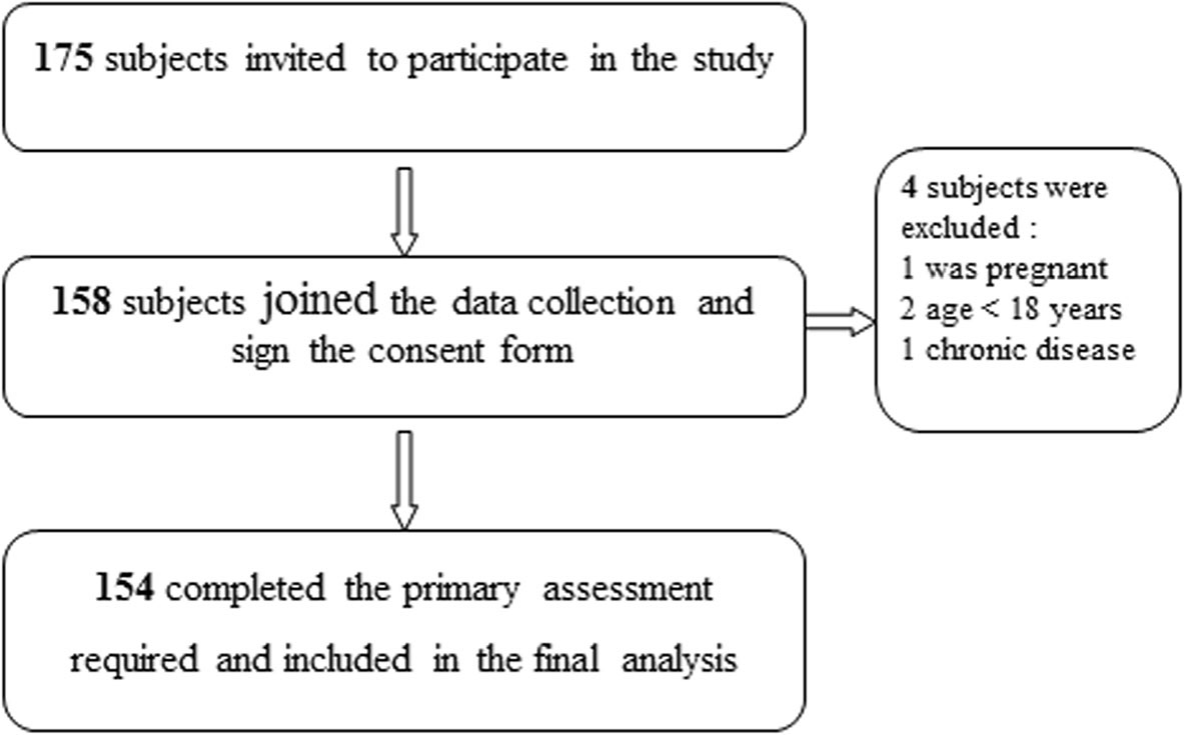
Subject recruitment flow chart

Subjects characteristics are presented in Table 2. A total of 154 females were included in the study. The mean age of the sample was (19.64 ± 1.170) and the mean of their academic achievment was (80.07 ± 7.130) out of 100. The procedure of recruiting females is described in Fig. 1.

**Table 2:** Subject characteristics presented in numbers and percentages n (%)

Variable		n	%
Faculties	Sciences	71	46.1
	Engineering and technology	39	25.3
	Human Sciences	44	28.6
Years of study	1st year	41	26.6
	2nd year	59	38.3
	3rd year	32	20.7
	(4 + 5) ^th^ year	22	14.3
State	Single	138	89.6
	Married + otherwise	16	10.4
Area of living	City	94	61.1
	Village + camp	60	38.9
Monthly Family income	< 3000	41	26.6
	3000–5000	77	50
	> 5000	36	23.4
Type of housing	With family	128	83.2
	University hostels	26	16.8

### Subjects’ body mass index

The results reveled that majority of the participants (68.1%) are considered normal weight, 9.2% underweight, 19.9% overweight and 2.8% are underweight.

### Prevalence of binge eating symptoms and its relationship with socio-demographic variables

Half of the participants (50%) showed positive binge eating symptoms. The relationships between binge and socio-demographic variables; area of living, marital status were not significant, similarly there were no significant relationship with faculties, years of study or academic achievement (*p* > 0.05).

### The relationship between body mass index, dietary habits with presence of binge symptoms

**Fig. 2:**
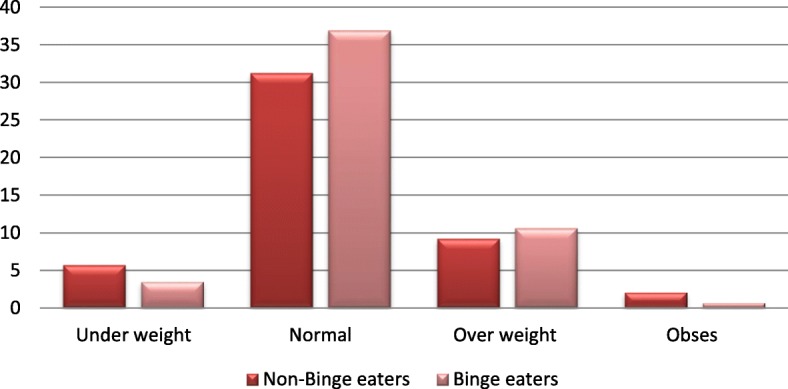
Association between binge eating symptoms and BMI. NS at *p* > 0.05 using Chi Square test

Figure 2 illustrates that there was no association between presence of binge eating symptoms and BMI using Chi square test. Moreover, the results show a higher prevalence of Binge eating symptoms is associated with eating between meals χ^2^ (1, *n* = 154, *p* value = 0.035) and number of snacks χ^2^ (1, n = 154, p value = 0.045), while it was not associated with meal skipping, eating fast food, eating alone or with family. Similarly, it was not associated with weight satisfaction.

### Relationship between presence of Binge Eating Symptoms & Dietary Intake

Table 3 demonstrates that there was no significant association between presence of binge eating symptoms and dietary intake (*p* < 0.05).

**Table 3:** The relationship between presence of binge eating symptoms and Dietary intake

	Unite	Binge eaters	Non-binge eaters	t	CI	*P* value
Total calories	Kcal /day	2709 ± 800	2410 ± 759	−2.72	− 511.2230.4	0.19
Carbohydrate	gm/day	335 ± 140	310 ± 146	−0.748	−69.5- 28.4	0.23
Protein	gm/day	105 ± 34	100 ± 42	−0.830	−23.3-9.66	0.11
Fat	gm/day	111 ± 46	104 ± 34	−0.817	−16.8- 10.31	0.204
Fiber	gm/day	19.5 ± 8	20 ± 9	−0.476	−2.25- 3.7	0.13
Sugar	gm/day	81 ± 42	78 ± 41	0.487	−11.1-17.08	0.53

N.S using independent t-test

### Relationship between presence of binge eating symptoms & psychosocial variables

Table 4 demonstrates that individuals with binge eating symptoms had significantly higher scores on depression, stress and anxiety that those without symptoms (*p* < 0.05).

**Table 4:** The relationship between presence of binge eating symptoms and psychosocial variables presented in mean ± sd

	Binge eaters	Non-binge eaters	T	CI	*P* value
Depression	7.8 ± 4.6	5.9 ± 3.8	4.21	0.501–3.254	0.007*
Stress	10.1 ± 4.5	8.3 ± 3.9	1.39	0.43–3.100	0.011*
Anxiety	8.6 ± 4.8	6.2 ± 3.9	8.81	0.99–2.88	0.002*

*significant *p* < 0.05 using independent t-test

## Discussion

The aim of this study was to determine the prevalence of binge eating symptoms and its relationship with selected variables (i.e. socio-demographics, nutritional status and dietary habits) among undergraduate students at Palestine Polytechnic University, and to investigate the presence of psychological symptoms in subjects with binge eating symptoms.

In the present study, the results reveal that the prevalence of binge eating symptoms among female university students (50%) was relatively higher than the rates obtained in Iceland (0.6%) [22], Netherland (2.3%) [23], Canada (0.7%) [24], USA (3.0%) [25], Italy (0.6%) [26]. This higher rate could be due to different tools used to define the presence of binge eating symptoms. In addition, they could be due to different study aims as this study aimed to examine the presence of binge eating symptoms rather than make a diagnosis of binge eating disorder.

Till this date, cultural theories concerning the influence of Western exposure on the risk of eating disorder have concentrated on factors specific to eating disorders, e.g., media influences, body image ideals, and peer and familial pressures on appearance. Moreover, the exposure to Western countries is correlated with high risk for a broad range of other psychiatric problems such as binge eating disorder [27]. The outcome of higher prevalence in this research could be assigned to media exposure which could be influencing opinions related to body weight and body image. Palestinian females who are not exposed to the Israeli society in a direct way still have the chance to be exposed to Israeli media through social media, television, and other means of communication. Lately, there has also been a growing exposure to Turkish media. Such TV shows and movies may also affect the attitudes and behaviors of Palestinian females, given that Turkish culture is not as conservative as Palestinian culture [14].

Even though obesity is linked with binge eating disorder, it is not included as a diagnostic criteria for binge eating disorder, which is distinguished from obesity [1]. Binge eating disorder is found across the body mass spectrum but is frequently found in individuals with obesity (36.2–42.4%) [28]. A small percentage of people attempting to lose weight were diagnosed with binge eating disorder (13%–27%) [28–30] In the current study, it was found that there is no association between weight status and binge eating symptoms. Unlike former research where it was noted that overweight/obesity was highly linked to binge eating among adolescents from a high SES population [31]. The difference may be because the current study examined binge eating symptoms rather than examining binge eating disorder diagnosis. This presence is alarming sign for developing the disorder which is associated with obesity and overweight.

The current research indicates that binge eating symptoms is significantly related to stress. This finding is supported by the liturature [32–36]. Since 1959, case reports of Stunkard elucidated that binge-eaters was experiencing marked distress. Elevated levels of distress associated with binge eating are stated in binge-eaters who have either normal weight or obesity, proposing that the distress is not an outcome of comorbid obesity [37]. The current outcomes also confirm that binge-eating is significantly correlated with anxiety, and this outcome is consistent by previous study performed by Jung and his colleagues [2].

In addition, we have noticed in the current study that there is significant association between binge eating and depression, and this result is consistent with former studies. For instance; Carriere and his colleagues have observed that subjective binge eating was significantly associated with depression [38]. In another study, it was stated that the more severe the depression, the more the severe the binging [39]. French and his colleagues stated that binge-eaters have higher levels of stressful life events and depression in comparison with non-binge eaters [40].

Our study reveals that binge eaters have a slightly distinct eating behavior beyond binge eating. As it was observed that binge-eaters consumed a slightly higher amount of total calories (300 kcal more), which is supported by the literature [41], and a higher amount of calories as a fat when compared to non-binge eaters, however, the differences are insignificant. Overall, little data is available about the eating habits of females who binge eat. Former studies have found few macronutrient variations among binge and non-binge eaters on regular intake at meals. But there is some evidence of elevated fat intake during binge episodes [42, 43]. In a laboratory setting, it was noticed that obese subjects with binge eating disorder consume more calories and a higher amount of calories as a fat than obese subjects without binge eating disorder [42, 43]. Our results also indicate that there are no differences between binge-eaters and non-binge eaters in the consumed amounts of carbohydrates, protein, sugar, and fiber. This finding is confirmed by former studies as well [42, 43].

There is a complexity in illustrating eating data among females who binge eat, since habitual patterns of eating are typically estimated. Episodes of binge eating may not be reflected by using regular dietary intake questionnaires, especially if the occurrence of binge is low. Furthermore, it is unclear how individuals are stating eating habits that surround their binge eating. For instance, do they integrate these eating episodes into their average habit reports, or are these episodes are excluded from the ‘average’ habit, because they are seen as uncommon and not representative of their usual patterns? Moreover, embarrassment or shame could result in the elimination of these episodes from self-reports of eating habit. Limited sources are available that resolve these methodological issues [40].

There are a few limitations in the current study. The study only included participants from one university which means that these results are not representative for females’ university students in Palestine. Nevertheless, the current study provides for the first-time worthy data on the prevalence of binge eating disorder in Palestine and its association with psychosocial variables.

## Conclusion

The present study reveals that the prevalence of binge eating symptoms is relatively high among female Palestinian university students. It was further demonstrated that there is no association between the disorder and body weight status. It also affirms that binge eating symptoms is associated with psychosocial factors such as depression, stress, and anxiety. Future research, that take into consideration a great number of psychological and demographic factors, is needed. According to this study, it is recommended to develop educational programs to increase the level of awareness regarding appropriate nutrition in relation to body weight, and it is possible that a general university optional course would be useful in this regard.

## Data Availability

Data and materials are available upon request and with permission of Dr. Manal Badrasawi at m.badrasawi@najah.edu.

## Abbreviations

AN: Anorexia nervosa
BN: Bulimia nervosa

## References

[1] American Psychiatric Association (2013). Diagnostic and statistical manual of mental disorders.

[2] Jung J-Y, Kim K-H, Woo H-Y, Shin D-W, Shin Y-C, Oh K-S, Shin E-H, Lim S-W (2017). Binge eating is associated with trait anxiety in Korean adolescent girls: a cross sectional study. BMC Womens Health.

[3] Brown JE, Lechtenberg E (2017). Nutrition through the life cycle.

[4] Dingemans A, Danner U, Parks M (2017). Emotion regulation in binge eating disorder: a review. Nutrients..

[5] Kessler RC, Berglund PA, Chiu WT, Deitz AC, Hudson JI, Shahly V, Aguilar-Gaxiola S, Alonso J, Angermeyer MC, Benjet C, Bruffaerts R, de Girolamo G, de Graaf R, Maria Haro J, Kovess-Masfety V, O'Neill S, Posada-Villa J, Sasu C, Scott K, Viana MC, Xavier M (2013). The prevalence and correlates of binge eating disorder in the World Health Organization world mental health surveys. Biol Psychiatry.

[6] Hutson PH, Balodis IM, Potenza MN (2018). Binge-eating disorder: clinical and therapeutic advances. Pharmacol Ther.

[7] Lee-Winn AE, Reinblatt SP, Mojtabai R, Mendelson T (2016). Gender and racial/ethnic differences in binge eating symptoms in a nationally representative sample of adolescents in the United States. Eat Behav.

[8] Hudson JI, Hiripi E, Pope HG, Kessler RC (2007). The prevalence and correlates of eating disorders in the National Comorbidity Survey Replication. Biol Psychiatry.

[9] Eisenberg D, Nicklett EJ, Roeder K, Kirz NE (2011). Eating disorder symptoms among college students: prevalence, persistence, correlates, and treatment-seeking. J Am Coll Heal.

[10] Wiederman MW, Pryor TL (2000). Body dissatisfaction, bulimia, and depression among women: the mediating role of drive for thinness. Int J Eat Disord..

[11] Javaras KN, Pope HG, Lalonde JK, Roberts JL, Nillni YI, Laird NM, Bulik CM, Crow SJ, McElroy SL, Walsh BT, Tsuang MT, Rosenthal NR, Hudson JI (2008). Co-occurrence of binge eating disorder with psychiatric and medical disorders. J Clin Psychiatry.

[12] Kornstein SG, Kunovac JL, Herman BK, Culpepper L (2016). Recognizing binge-eating disorder in the clinical setting: a review of the literature. Prim Care Companion CNS Disord.

[13] Araujo DM, Santos GF, Nardi AE (2010). Binge eating disorder and depression: a systematic review. World J Biol Psychiatry.

[14] Saleh RN, Salameh RA, Yhya HH, Sweileh WM (2018). Disordered eating attitudes in female students of an-Najah National University: a cross-sectional study. J Eat Disord.

[15] Musaiger AO, Al-Mannai M, Tayyem R, Al-Lalla O, Ali EYA, Kalam F, Benhamed MM, Saghir S, Halahleh I, Djoudi Z, Chirane M (2013). Risk of disordered eating attitudes among adolescents in seven Arab countries by gender and obesity: a cross-cultural study. Appetite..

[16] Bartlett JE, Kotrlik JW, Higgins CC (2001). Organizational research: determining appropriate sample size in survey research appropriate sample size in survey research. Inf Technol Learn Perform J.

[17] Herman BK, Deal LS, DiBenedetti DB, Nelson L, Fehnel SE, Brown TM (2016). Development of the 7-item binge-eating disorder screener (BEDS-7). Prim Care Companion CNS Disord..

[18] Lee RD, Nieman DC (2013). Nutritional assessment.

[19] WHO. Body mass index – report. 2018. http://www.euro.who.int/en/health-topics/disease-prevention/nutrition/a-healthy-lifestyle/body-mass-index-bmi. Assessed 15 May 2019.

[20] Hamdan M, Monteagudo C, Lorenzo-Tovar M-L, Tur J-A, Olea-Serrano F, Mariscal-Arcas M (2014). Development and validation of a nutritional questionnaire for the Palestine population. Public Health Nutr.

[21] Moussa MT, Lovibond P, Laube R, Megahead HA (2017). Psychometric properties of an Arabic version of the depression anxiety stress scales (DASS). Res Soc Work Pract.

[22] Thorsteinsdottir G, Ulfarsdottir L (2008). Eating disorders in college students in Iceland. Eur J Psychiat.

[23] Smink FRE, van Hoeken D, Oldehinkel AJ, Hoek HW (2014). Prevalence and severity of DSM-5 eating disorders in a community cohort of adolescents. Int J Eat Disord..

[24] Flament MF, Buchholz A, Henderson K, Obeid N, Maras D, Schubert N, Paterniti S, Goldfield G (2015). Comparative distribution and validity of DSM-IV and DSM-5 diagnoses of eating disorders in adolescents from the community. Eur Eat Disord Rev.

[25] Stice E, Marti CN, Rohde P (2013). Prevalence, incidence, impairment, and course of the proposed DSM-5 eating disorder diagnoses in an 8-year prospective community study of young women. J Abnorm Psychol.

[26] Favaro A, Ferrara S, Santonastaso P (2003). The Spectrum of eating disorders in young women. Psychosom Med.

[27] Swanson SA, Saito N, Borges G, Benjet C, Aguilar-Gaxiola S, Medina-Mora ME, Breslau J (2012). Change in binge eating and binge eating disorder associated with migration from Mexico to the US. J Psychiatr Res.

[28] Barnes RD, White MA, Martino S, Grilo CM (2014). A randomized controlled trial comparing scalable weight loss treatments in primary care. Obesity (Silver Spring).

[29] Kolotkin RL, Westman EC, Østbye T, Crosby RD, Eisenson HJ, Binks M (2004). Does binge eating disorder impact weight-related quality of life?. Obes Res.

[30] Sandberg RM, Dahl JK, Vedul-Kjelsås E, Engum B, Kulseng B, Mårvik R, Eriksen L (2013). Health-related quality of life in obese Presurgery patients with and without binge eating disorder, and subdiagnostic binge eating disorders. J Obes.

[31] West CE, Goldschmidt AB, Mason SM, Neumark-Sztainer D (2019). Differences in risk factors for binge eating by socioeconomic status in a community-based sample of adolescents: findings from project EAT. Int J Eat Disord..

[32] Bentley C, Gratwick-Sarll K, Harrison C, Mond J (2015). Sex differences in psychosocial impairment associated with eating disorder features in adolescents: a school-based study. Int J Eat Disord..

[33] Colles SL, Dixon JB, O’Brien PE (2008). Loss of control is central to psychological disturbance associated with binge eating disorder. Obesity (Silver Spring).

[34] Mitchell KS, Neale MC, Bulik CM, Aggen SH, Kendler KS, Mazzeo SE (2010). Binge eating disorder: a symptom-level investigation of genetic and environmental influences on liability. Psychol Med.

[35] Striegel-Moore RH, Dohm FA, Solomon EE, Fairburn CG, Pike KM, Wilfley DE (2000). Subthreshold binge eating disorder. Int J Eat Disord..

[36] Stunkard AJ (1959). Eating patterns and obesity. Psychiatr Q.

[37] Goldschmidt AB, Le Grange D, Powers P, Crow SJ, Hill LL, Peterson CB, Crosby RD, Mitchell JE (2011). Eating disorder symptomatology in Normal-weight vs. obese individuals with binge eating disorder. Obesity (Silver Spring).

[38] Carriere C, Michel G, Féart C, Pellay H, Onorato O, Barat P, Thibault H (2019). Relationships between emotional disorders, personality dimensions, and binge eating disorder in French obese adolescents. Arch Pediatr.

[39] Berkowitz R, Stunkard AJ, Stallings VA (1993). Binge-eating Disorder in Obese Adolescent Girls. Ann N Y Acad Sci.

[40] French S, Jeffery R, Sherwood N, Neumark-Sztainer D (1999). Prevalence and correlates of binge eating in a nonclinical sample of women enrolled in a weight gain prevention program. Int J Obes Relat Metab Disord.

[41] Vannucci A, Tanofsky-Kraff M, Crosby RD, Ranzenhofer LM, Shomaker LB, Field SE, Mooreville M, Reina SA, Kozlosky M, Yanovski SZ, Yanovski JA (2013). Latent profile analysis to determine the typology of disinhibited eating behaviors in children and adolescents. J Consult Clin Psychol.

[42] Raymond NC, Bartholome LT, Lee SS, Peterson RE, Raatz SK (2007). A comparison of energy intake and food selection during laboratory binge eating episodes in obese women with and without a binge eating disorder diagnosis. Int J Eat Disord.

[43] Yanovski SZ, Leet M, Yanovski JA, Flood M, Gold PW, Kissileff HR, Walsh BT (1992). Food selection and intake of obese women with binge-eating disorder. Am J Clin Nutr.

